# Solution-Processed Yellow Organic Light-Emitting Diodes Based on Two New Ionic Ir (III) Complexes

**DOI:** 10.3390/molecules27092840

**Published:** 2022-04-29

**Authors:** Chaoxiong Guo, Song Guo, Qiqing Lu, Zizhan Jiang, Yuzhen Yang, Weiqiao Zhou, Qin Zeng, Jun Liang, Yanqin Miao, Yuanli Liu

**Affiliations:** 1 Guangxi Key Laboratory of Optical and Electronic Materials and Devices, College of Materials Science and Engineering, Guilin University of Technology, Guilin 541004, China; 1020190104@glut.edu.cn (C.G.); ahguosong@163.com (S.G.); jiangzizhan2021@163.com (Z.J.); yangyuzhen812@163.com (Y.Y.); wangyi123zhou@163.com (W.Z.); zqinnn@163.com (Q.Z.); 2 MOE Key Laboratory of Interface Science and Engineering in Advanced Materials, Taiyuan University of Technology, Taiyuan 030024, China; cat2675639894@163.com

**Keywords:** ionic iridium (III) complexes, organic light-emitting diodes, yellow phosphorescence, solution-process

## Abstract

Two new and efficient cationic yellow-emissive Ir (III) complexes (Ir1 and Ir2) are rationally designed by using 2-(4-chloro-3-(trifluoromethyl)phenyl)-4-methylquinoline as the main ligand, and, respectively, 4,4′-dimethyl-2,2′-bipyridyl and 4,4′-dimethoxy-2,2′-bipyridyl as the ancillary ligands. Both complexes show enhanced phosphorescence (546 nm with 572 nm as shoulder and high phosphorescent quantum efficiency in solution, which is in favor of efficient solution-processed phosphorescent organic light-emitting diodes. Compared with Ir2, the Ir1-based device displays excellent device performance, with maximum external quantum efficiency, current efficiency, and power efficiency of up to 7.92%, 26.32 cd/A and 15.31 lm/W, respectively, thus proving that the two new ionic Ir (III) complexes exhibit great potential for future solution-processed electroluminescence.

## 1. Introduction

In recent decades, organic light-emitting diode (OLED) has emerged as a very promising technology for lighting and display owing to its distinct advantages, such as flexible machining, large-area production, self-luminosity, low power consumption, etc. [1,2,3,4,5,6,7,8]. The materials used in OLEDs, especially in the emissive layer (EML), are crucial for device performance. To obtain highly efficient OLEDs, transition-metal complexes, particularly Ir (III) complexes, have received considerable attention due to their unique photophysical characteristics, for instance, tunable emission wavelength, rich excited states, and good photostability [9,10,11]. Furthermore, the heavy atom effect can promote intersystem crossing from the singlet state to the triplet state. In OLEDs based on Ir (III) complexes, both the singlet and triplet excitons can be harnessed simultaneously, thereby achieving 100% theoretical internal quantum efficiency. Since yellow is an important chromaticity element in the region of visible light, developing high-performance yellow OLEDs has a significant practical value [12,13,14].

In 2021, Zheng et al. integrated carbazole unit and neutral Ir (III) complexes to facilitate hole injection/transport, charge balance, and achieve high-performance OLEDs via vacuum evaporation with maximum luminance (L_max_), maximum EQE (EQE_max_) and maximum power efficiency (PE_max_) of 40,624 cd m^−2^, 32.2%, and 71.7%, respectively [15]. In 2019, Yang and Ma reported a neutral phosphorescent Ir (III) complex using 3-(2,4-difluorophenyl)-6-methylpyridazine as the main ligand, this complex possessed a small steric hindrance of the chelating nitrogen atom, leading to a strong coordination bond between the chelating nitrogen atom and metal iridium atom, which is beneficial for the stability and luminescent efficiency. The yellow OLED based on this Ir (III) complex fabricated via vacuum evaporation showed good performance with EQE_max_ of 22.7%, L_max_ of 26,186 cd m^−2^, and PE_max_ of 84.2% [16]. However, most phosphorescent yellow emissive Ir (III) complexes used in OLEDs are based on neutral complexes, because of their low sublimation point, which is suitable for vacuum sublimation to obtain good film-forming property [17,18,19,20,21,22]. Nevertheless, this type of processing method will consume a lot of rare metals and energy. Compared with the neutral phosphors, ionic Ir (III) complexes have their own unique advantages. For instance, the auxiliary ligands of the ionic Ir (III) complexes possess more diverse selectivity and are easy to be modified and synthesized [23,24]. In 2007, Wong’s group reported two sublimable cationic yellow emissive Ir (III) complexes with a large cyclometalating ligand to prepare OLEDs by the method of vacuum evaporation with L_max_ and EQE_max_ of 15,610 cd m^−2^ and 7%, respectively, demonstrating that ionic Ir (III) materials would be a useful alternative electric-luminogen for evaporated OLEDs [25]. Qiu’s group developed a versatile strategy to regulate the sublimation ability of ionic Ir (III) complex by introducing counterion with large steric hindrance. Based on this strategy, they designed a series of cationic Ir (III) complexes, which were successfully used to fabricate multi-colored phosphorescent OLEDs by vacuum evaporation, achieving a peak EQE of 8.1% [26,27]. Very recently, Wong’s group developed a yellow cationic Ir (III) complex with large steric hindrance of counterion using the above strategy, which possessed a full width at half maximum (FWHM) of 105 nm; this was then utilized to fabricate yellow and white OLED via vacuum evaporation, obtaining the peak EQE of 11.6% [28]. Although tremendous efforts have contributed to developing ionic Ir (III) complexes for the highly efficient solution-processable OLEDs, it is still an arduous assignment because of the lack of versatility of these phosphors. Consequently, it is of great significance to explore more ionic Ir (III) complexes in this field.

Herein, we selected a previously reported ligand 2-(4-chloro-3-(trifluoromethyl)phenyl)-4-methylquinoline as the cyclometalating ligand, 4,4′-dimethyl-2,2′-bipyridyl and 4,4′-dimethoxy-2,2′-bipyridyl as the ancillary ligands, and the commonly used small ion PF_6_^−^ was served as the counterion, obtaining two cationic Ir (III) complexes (Ir1 and Ir2). These both exhibited similar photophysical properties and bright yellow emission with high photoluminescent quantum efficiency, which were further utilized to fabricate solution-processed OLEDs. Notably, the auxiliary ligands substituted by methyl and methoxy group showed great influence on the device performances. Complex Ir1 substituted by methyl showed an EQE_max_, current efficiency (CE) and PE_max_ of 7.92%, 26.32 cd/A and 15.31 lm/W, respectively. On the contrary, complex Ir2 showed inferior device performances.

## 2. Results

### 2.1. Structural Characterization

**Figure 1:**
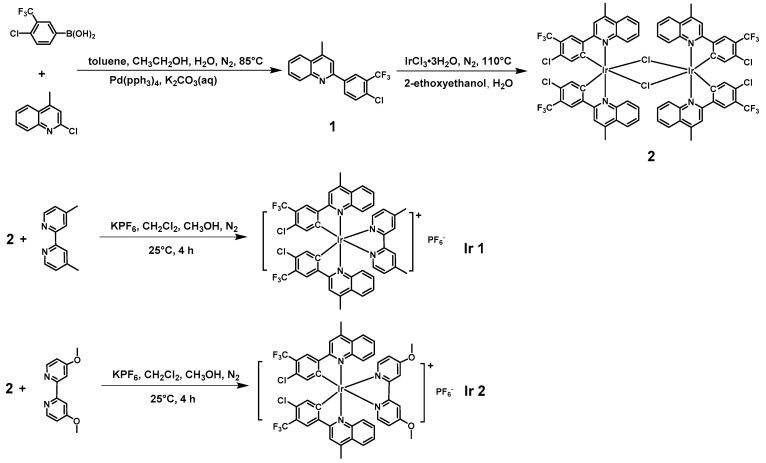
Synthetic routes of Ir1 and Ir2.

Two complexes were synthesized according to Figure 1. First, the main ligand 2-(4-chloro-3-(trifluoromethyl)phenyl)-4-methylquinoline was obtained via Suzuki coupling reaction with the aid of Pd(0) catalyst. Then, μ-dichloro bridged Ir (III) dimer complex and the product Ir1 and Ir2 were synthesized using the methods reported in previous literature with appreciable yields. The structures of the intermediates and end-products were verified via ^1^H NMR (500 MHz), ^13^C NMR (125 MHz), ^31^P NMR (202 MHz), ^19^F NMR (470 MHz) spectra and mass spectra. The photophysical properties of the complexes were characterized via UV/vis absorption spectrometry, and steady-state and transient phosphorescence spectrometry. In the ^31^P NMR and ^19^F NMR, the coupling between ^31^P and ^19^F can be clearly observed from the heptet and doublet peaks, respectively, as shown in the Supporting Information.

### 2.2. Photophysical Properties

**Figure 2:**
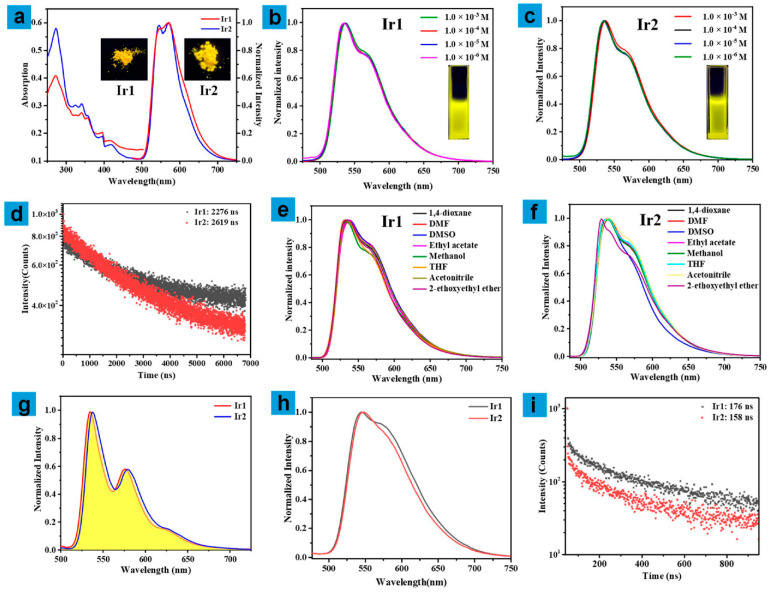
(**a**) Absorption spectra (left) and emission spectra (right) of Ir1 (red line) and Ir2 (blue line) in dichloromethane in solid state, inset: the photographs of Ir1 and Ir2 in solid-state excited at 365 nm, (**b**,**c**) emission spectra of Ir1 and Ir2 at different concentrations in dichloromethane at room temperature, inset: the photograph of Ir1 and Ir2 in dichloromethane excited at 365 nm (1.0 × 10^−3^ M), (**d**) The decay curves of phosphorescent lifetime of Ir1 and Ir2 in CH_2_Cl_2_ solution, (**e**,**f**) emission spectra of Ir1 and Ir2 in different solvents at the concentration of 1.0 × 10^−5^ M, (**g**) the low-temperature emission spectrum of Ir1 and Ir2 at 77 K in 2-MeTHF. (**h**) emission spectra of Ir1 and Ir2 at neat film state, (**i**) The decay curves of phosphorescent lifetime of Ir1 and Ir2 in neat film state.

Firstly, the photophysical properties of the two complexes were measured, as shown in Figure 2. The absorption spectra of Ir1 and Ir2 in CH_2_Cl_2_ at the concentration of 1.0 × 10^−5^ mol/L (Figure 2a) exhibited almost the same peak pattern. The absorption peak at 273 nm can be ascribed to the ligand-centered (LC) transition (π-π*) of the main ligand. The relatively weak band peaking at 330–420 nm can be ascribed to a mixture of the spin-allowed singlet metal-to-ligand charge transfer (^1^MLCT) and ligand-to-ligand charge transfer (^1^LLCT) transitions [29,30,31].

The phosphorescence behaviors of Ir1 and Ir2 in the solid-state were then studied. Both of these exhibited bright yellow emission to the naked eye at the excitation of 365 nm (Figure 2a, inset), and their phosphorescent spectra showed a similar profile, both peaking at 546 nm and 572 nm (shoulder). The emission lifetime and full width at half maximum (FWHM) of Ir1 and Ir2 are 362/369 ns (signal exponential function) and 93/80 nm, respectively (Figure S1). Next, the emission spectra of Ir1 and Ir2 in degassed dichloromethane with different concentrations from 1.0 × 10^−3^ M to 1.0 × 10^−6^ M (Figure 2b,c) were measured, both of which exhibited a maximum emission peak at 535 nm and a shoulder peak at 569 nm, respectively, with a modest FWHM of 75 nm. The results reveal that there was almost no intermolecular interaction between the complex molecules in the dilute solution. The lifetimes of Ir1 and Ir2 at the peak of 535 nm are 2.2 and 2.6 μs, respectively, the decay curves (Figure 2d) exhibited signal exponential function, confirming their triplet-emitting nature [32]. Meanwhile, Ir1 and Ir2 both exhibited high phosphorescent quantum efficiency of 0.89 and 0.87, respectively. The emission spectra of the two complexes in different solvents (1,4-dioxane, 2-ethoxyethyl ether, methanol, acetonitrile, tetrahydrofuran, N,N-dimethyl formamide, dimethylsulfoxide, and ethyl acetate) of bubbled N_2_ were carried out as illustrated in Figure 2e,f, which suggests that the maximum emission peak changed about 10 nm along with the alteration of the polarity of solvents. For example, the maximum emission peaks of Ir1 in DMF and DMSO were 529 and 538 nm, respectively, while the maximum emission peaks of Ir2 in 2-ethoxyethyl ether and acetonitrile were 529 and 538 nm, respectively. The shoulder peak remained unchanged. The slight shift indicates that the phosphorescent emission may occur from a mixture of the LC excited state and metal-to-ligand charge transfer (MLCT) [33].

The low temperature (77 K) spectra of Ir1 and Ir2 in 2-methyltetrahydrofuran (2-MeTHF) were measured, as depicted in Figure 2g, and they both demonstrated obvious fine structures of the vibronic bands. Ir1 peaked at 535, 576, and 625 nm, respectively. Ir2 showed a very slight bathochromic shift by 3 nm, peaking at 538, 580, and 628 nm, respectively. The triplet level of Ir1 and Ir2 can be estimated to be 2.32 and 2.30 eV, respectively, via the highest-energy vibronic band in the spectra [33].

Because complexes Ir1 and Ir2 are used in OLEDs as thin films, their photophysical properties are presented in solid neat thin films. The phosphorescent spectra (Figure 2h) of the two compounds exhibited similar profiles, located at 545 and 575 nm (shoulder) with FWHM of 98 nm (Ir1) and 90 nm (Ir2). It is worth noting that compared with the spectral shape of the three states (solid-state, solution and neat film), the signal of the shoulder peak decreased from solid-state to a solution with a reduction of 23%, and the relative intensity of the signal of shoulder peak in the neat film is between the other two states. This phenomenon is very common in the literature [34,35], which may be due to the partial conjugation between the isoquinoline moiety and the chromophore core [36]. The lifetimes of Ir1 and Ir2 are 176 and 158 ns, respectively, which are smaller than those in the solution state. The decay curves (Figure 2i) of the two compounds displayed signal exponential function, demonstrating that the corresponding lifetime can be assigned to the excited states [32]. Besides, compared with the solution state, the phosphorescent quantum efficiency of the two neat films (0.17 for Ir1, and 0.14 for Ir2) is relatively low, which may be due to the triplet-triplet annihilation. The photophysical data of Ir1 and Ir2 are summarized as shown in Table 1. Photostability is a very important indicator for materials used in OLEDs. To investigate this indicator for Ir1 and Ir2, the solutions of the two complexes at the concentration of 1.0 × 10^−5^ M were prepared and excited by 365 nm lamp (20 mW/cm^2^) for 30 min continuously, and the phosphorescent intensity at 535 nm was recorded, respectively, as shown in Figure S2. The almost constant emissive intensity revealed excellent photostability of Ir1 and Ir2, suitable for fabricating the devices. Meanwhile, the cyclic voltammetry curves of Ir1 and Ir2 were carried out, as shown in Figure S3, and they both exhibited an irreversible oxidation wave at 1.74 V, which may be ascribed to the oxidation of the iridium center [37]. In general, there is almost no significant differential influence of the ancillary ligand in solid and solution state for the photoluminescence of Ir1 and Ir2.

### 2.3. Mechanism Analysis

**Figure 3:**
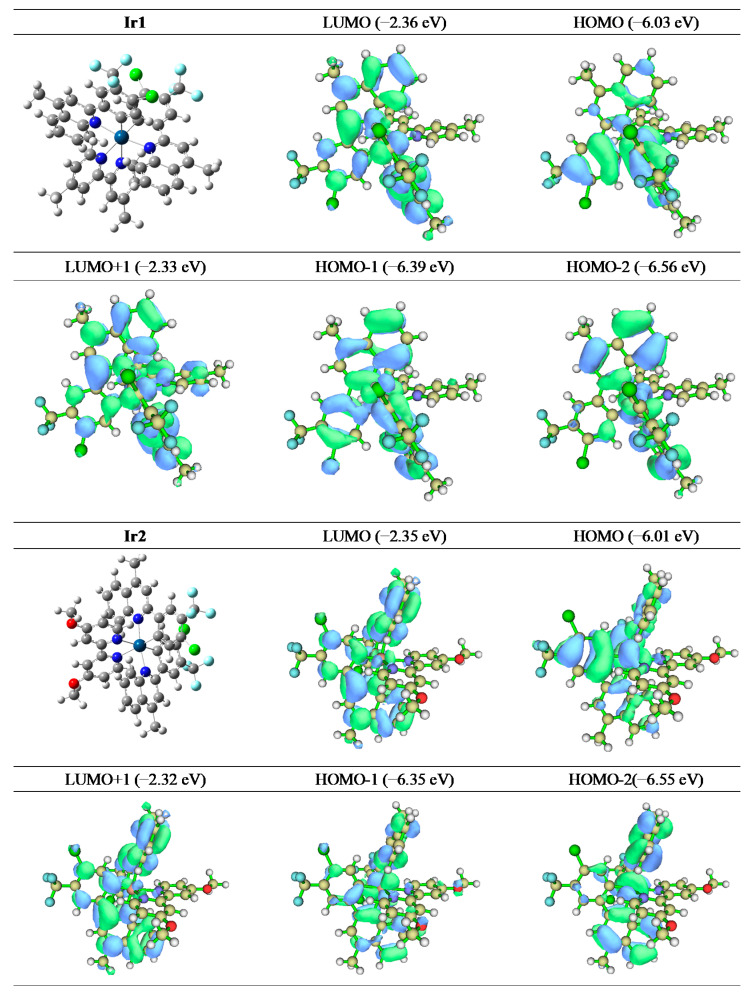
The distributions of molecular orbitals of Ir1 and Ir2.

To understand the luminescent behaviors of Ir1 and Ir2 in depth, theoretical calculations were performed by means of B3LYP and time-dependent density functional theory (TDDFT), as shown in Figure 3 and Table 2. The highest occupied molecular orbital (HOMO) and the lowest unoccupied molecular orbital (LUMO) energy levels of Ir1 and Ir2 were −6.03/−2.36 eV and −6.01/−2.35 eV, respectively. From the optimized spatial configuration of Ir1 and Ir2, we can clearly see that both complexes adopted twisted octahedral configuration, resulting in weak intermolecular interaction, coincident with the similar outline of the emission spectra in the dilute solution at different concentrations. The frontier molecular orbitals (FMO) indicate that, different from the usual Ir (III) complexes, the HOMO and LUMO of both Ir1 and Ir2 were located on the cyclometalating ligand and iridium center without the auxiliary ligand. Meanwhile, HOMO−1, LUMO+1 and HOMO−2 of Ir1 and LUMO+1, HOMO−1 and HOMO−2 of Ir2 are also both located on the cyclometalating ligand and iridium center. The excited states of Ir1 and Ir2 could be ascribed to a mixture of MLCT transition and LLCT transition. Additionally, the auxiliary ligand was not involved in frontier molecular orbital construction, which can well explain the similar or even identical photophysical behaviors of the two complexes [38,39].

### 2.4. Electroluminescent Devices

**Figure 4:**
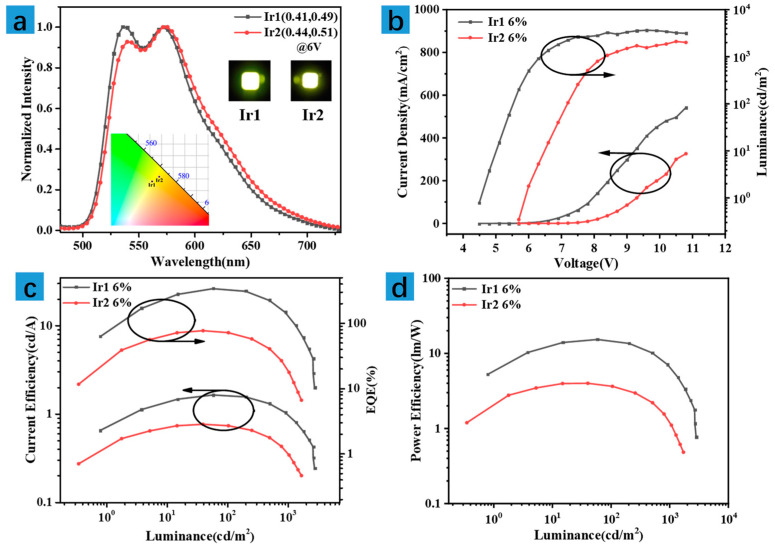
(**a**) The normalized EL spectra, (**b**) the J-V-L curves, (**c**) CE-L-EQE curves, and (**d**) PE-L curves of the yellow OLEDs based on Ir1 and Ir2 with the doping concentration of 6% in CBP. Inset: EL photographs of OLEDs under the voltage of 6 V.

To investigate the electroluminescent performances of the two cationic complexes, Ir1 and Ir2 were used to prepare the yellow phosphorescent OLEDs through solution process. The device structure was depicted as follows: indium tin oxide (ITO)/poly(3,4-ethylenedioxythiophene) doped with poly-styrenesulfonate (PEDOT:PSS) (40 nm)/emissive layer (20 nm)/1,3,5-Tris(1-phenyl-1*H*-benzimidazol-2-yl)benzene (TPBi) (50 nm)/LiF (1 nm)/Al (100 nm). ITO, PEDOT:PSS and TPBi acted as the transparent anode, hole-injection layer, and electron-transporting layer, respectively. The emissive layer adopted 4,4′-Bis(N-carbazolyl)-1,1′-biphenyl (CBP, a frequently used host material) mixed with Ir1 or Ir2 with a mass ratio of 6%, respectively. The major electroluminescence behaviors could be ascribed to robust carrier harvesting in the mixture of host and guest. Moreover, LiF and Al were utilized as cathode. The corresponding device performances based on ionic Ir1 and Ir2 are shown in Figure 4. Figure 4a is the electroluminescence spectra of Ir1 (gray line) and Ir2 (red line), which both exhibited dual-emission peaking at 537/572 (Ir1) and 540/574 nm (Ir2), respectively, and FWHM of 92 (Ir1) and 100 nm (Ir2). The subtle change of the emission spectra may be attributed to the microcavity effect of the devices. The inserts are the photographs of the devices based on Ir1 and Ir2, and they all exhibited bright yellow emission with the 1931 Commission Internationale de L’Eclairage (CIE) coordinates of (0.41, 0.49) and (0.44, 0.51), respectively. Notably, although the emission spectra of the two complexes are nearly identical, the device performance varied greatly. The turn-on voltages at 1 cd/m^2^ of the devices based on Ir1 and Ir2 were 4.5 and 6.0 V, respectively. The low turn-on voltage of Ir1 suggested relatively poor injection barriers between the electron-transporting layer and emissive layer. The L-V-J curves of the yellow devices are depicted in Figure 4b. The key parameters of the devices were listed in Table 3. The maximum luminance of Ir1 and Ir2 are 3662 and 2106 cd/m^2^, respectively. The maximum CE, PE, and EQE of the yellow OLEDs based on Ir1 and Ir2 are 26.32/8.78 cd/A, 15.31/4.00 lm/W, and 7.92/2.81%, respectively. 

Compared with the Ir1-based device, the OLED based on Ir2 exhibited inferior performance. To understand the reasons for this phenomenon in depth, considering that the formation of the emissive layer needs to be annealed for 10 min at 60 °C after the spin-coating, thermal stability for the structure is an important evaluation index. Thermogravimetric analysis (TGA) of Ir1 and Ir2 was carried out, as shown in Figure S5. The results implied that Ir1 and Ir2 showed decreased temperatures (Td: 5% weight loss) of 323 and 247 °C, respectively, suggesting that Ir2 possessed relatively poor thermal stability. Then, the thermal stability of the phosphorescent behaviors of the two complexes was measured, as shown in Figure S6. The results showed that compared with the phosphorescent spectra at room temperature (298 K), the intensity of the peak at 575 nm decreased by about 4.8% for Ir1 and 5.9% for Ir2 at 330 K, respectively, and that of Ir2 decreased dramatically with the continual increase in temperature, which is consistent with the TGA results. Thus, the poor thermal stability of both structure and phosphorescent behaviors of complex Ir2 may be responsible for its inferior electroluminescent performance.

Although the device performance of Ir1 is not optimal compared to the reported yellow OLEDs based on Ir (III) complexes, it is better than the mean level of the yellow devices based on the solution-processable ionic Ir (III) complexes. The results revealed that cationic Ir1 is an appropriate candidate for preparing novel solution-processed, high-efficiency OLEDs.

## 3. Materials and Methods

All operations were completed under an N_2_ atmosphere using standard Schlenk unless otherwise stated. All solvents were used after distillation and stored with molecular sieves. All reagents and intermediates were purchased by commercial sources and used without further purification. 

The detailed instrument models are mentioned in the Supplementary Materials [40,41].

All OLEDs were fabricated on ITO glass substrates with a sheet resistance of <15 Ω/sq. The ITO substrates were cleaned first with acetone, deionized water and acetone, and then dried in drying cabinet, and treated with ultraviolet-ozone for 20 min. Next, the substrates were dried under 50 °C. Before the experiments, substrates were treated with UV-O3 for 30 min to enhance the work function of ITO. PEDOT:PSS layers were spin-coated on the ITO substrates with the speed of 3000 rpm for 75 s and then annealed at 120 °C for 15 min. The emission layer (CBP:6%Ir2) was prepared by spin-coating (solvent: chloroform) at 5000 rpm for 60 s and annealed for 10 min at 60 °C in the glove box. The thickness of the emission layer is about 40 nm. Next, a 50 nm thickness TPBi, acting as electron-transporting layer, was deposited on the emission layer. Deposition rate of organic materials was 0.1 nm/s and vacuum pressure for thermal deposition was 5 × 10^−4^ Pa. After deposition of the TPBi layer, a cathode consisting of 1.0 nm LiF and 100 nm Al was deposited at a rate of 0.01 nm/s for LiF and 0.1–0.4 nm/s for Al. The thickness of the films was controlled by a quartz thickness monitor. The emission area of the devices is 12 mm^2^. The current-voltage-luminescence characteristics were measured by a Keithley 2602 source meter with a calibrated silicon photodiode. The electroluminescence spectra of the devices were analyzed with a spectrometer (PR655). The thickness of organic thin films was measured using a spectroscopic ellipsometry (α-SE, J.A. Wollam Co., Inc., Lincoln, NE, USA). All the samples were measured directly after fabrication without encapsulation in ambient atmosphere at room temperature.

## 4. Conclusions

In summary, two new cationic yellow-emitting Ir (III) complexes, namely Ir1 and Ir2, have been designed and synthesized by utilizing methyl and methoxyl-bearing auxiliary ligands. Both Ir1 and Ir2 showed bright yellow photoluminescence in the solid and solution state, which were further used to prepare the solution-processed OLEDs. Compared with Ir2, the Ir1-based device exhibited better performance with the maximum luminance, CE, PE, and EQE of 3662 cd/m^2^, 26.32 cd/A, 15.31 lm/W, and 7.92/%, respectively. The relatively high performance verified that the cationic Ir1 was an outstanding candidate for solution-processed OLED. Additionally, we will explore more ionic solution-processable Ir (III) complexes for high-quality OLEDs, and the optimization of the device structure is also an ongoing project in our lab.

## Figures and Tables

**Table 1:** Photophysical data of Ir1 and Ir2.

Complexes	Emission in CH_2_Cl_2_			Emission in Neat film			E_g_ [eV] ^a^	E_onset_^ox^ [eV]	T_1_ [eV] ^b^
	λ_em_ [nm]	τ [μs]	Φ_PL_	λ_em_ [nm]	τ [μs]	Φ_PL_			
Ir1	546,572	2.2	0.89	546,572	0.16	0.17	2.61	1.74	2.32
Ir2	546,572	2.6	0.87	547,572	0.10	0.16	2.61	1.74	2.30

^a^. E_g_ was estimated from absorption onset from UV-visible spectra; ^b^. T_1_ = 1240/λ_77K_.

**Table 2:** The theoretical calculation of molecular orbitals of Ir1 and Ir2.

Complex	State	HOMO/eV	LUMO/eV	Configuration	Character
Ir1	T_1_	−6.03	−2.36	HOMO-1 → LUMO, 33.8% HOMO → LUMO+1, 25.0% HOMO-2 → LUMO+1, 13.2%	LLCT MLCT/LLCT MLCT/LLCT
Ir2	T_1_	−6.01	−2.35	HOMO-1 → LUMO, 33.2%	MLCT/LLCT
				HOMO → LUMO+1, 31.8%	MLCT/LLCT
				HOMO-2 → LUMO+1, 16.2%	MLCT/LLCT

**Table 3:** EL data for the yellow OLEDs based on Ir1 and Ir2.

Complex	X%	λ_EL_/nm	CIE(x,y)	V_ON_/V	L_max_/cd⋅m^−2^	CE_max_/cd⋅A^−1^	PE_max_/lm⋅W^−1^	EQE/%	FWHM/nm
Ir1	6	537/572 (sh)	(0.41, 0.49)	4.5	3662	26.32	15.31	7.92	92
Ir2	6	540/574 (sh)	(0.44, 0.51)	6.0	2106	8.78	4.00	2.81	100

## Data Availability

Raw data are available from the authors upon request.

## Supplementary Materials

The following supporting information can be downloaded at: https://www.mdpi.com/article/10.3390/molecules27092840/s1, Figure S1: The decay curves of phosphorescent lifetime for Ir1 and Ir2 in solid state; Figure S2: The photostability of Ir1 and Ir2 excited at 365 nm in CH_2_Cl_2_ solution; Figure S3: The cyclic voltammograms of Ir1 and Ir2 under a scan rate of 100 mV/s in CH_2_Cl_2_; Figure S4: Schematic diagram of device structure (a) of the yellow OLEDs and chemical structures (b) of the materials involved in the prepared devices; Figure S5. TGA curves of Ir1 and Ir2; Figure S6: The emission spectra of Ir1 and Ir2 at different temperatures.

## Sample Availability

Samples of the compounds are not available from the authors.
