# Supplementary material for: Solution-Processed Yellow Organic Light-Emitting Diodes Based on Two New Ionic Ir (III) Complexes
Source: Molecules. 2022 Apr 29;27(9):2840. doi: 10.3390/molecules27092840 (PMC9101346; doi:10.3390/molecules27092840)
Supplement: Supplementary file 1 [file molecules-27-02840-s001.zip › molecules-1688448-Supplementary Material.pdf]

Supporting Information

Solution-Processed Yellow Organic Light-Emitting Diodes Based on Two New Ionic Ir(III) Complexes

Chaoxiong Guo[1,#], Song Guo1[,#], Qiqing Lu[2], Zizhan Jiang[1], Yuzhen Yang[1], Weiqiao Zhou[1], Qin Zeng[1], Jun Liang[1,*], Yanqin Miao[2,*], and Yuanli Liu[1,*]

[1] Guangxi Key Laboratory of Optical and Electronic Materials and Devices, College of Materials Science and Engineering, Guilin University of Technology, Guilin 541004, China; 1020190104@glut.edu.cn (C.G.); ahguosong@163.com (S.G.); jiangzizhan2021@163.com(Z.J.); yangyuzhen812@163.com(Y.Y.); wangyi123zhou@163.com(W.Z.); zqinnn@163.com(Q.Z.);

[2] MOE Key Laboratory of Interface Science and Engineering in Advanced Materials, Taiyuan University of Technology, Taiyuan 030024, PR China. cat2675639894@163.com (Q.L.)

* Correspondence: liangjun@glut.edu.cn(J.L.), lyuanli@glut.edu.cn (Y.L.), miaoyanqin@tyut.edu.cn (Y.M.)

# Chaoxiong Guo and Song Guo contributed equally to this work.

1. General Experimental Information

The NMR spectra were measured with a Bruker spectrometer at room temperature. Mass spectra were obtained on Bruker matrix-assisted laser desorption/ionization time-of-flight mass pectrometer (MALDI-TOF-MASS). The UV-vis absorption spectrum was recorded on a Lambda 750 spectrometer (PerkinElmer, America). Emission spectra and lifetimes were measured on a FluoroMax-4 fluorescence spectrophotometer (Horiba, Japan). Photographs were taken by Canon 90D camera (Canon, Japan). The absolute quantum yields of the complexes was determined through an absolute method by employing an integrating sphere. Cyclic voltammetry curves were accomplished in CH_2Cl_2 with a three-electrode cell configuration consisting of platinum working and counter electrodes and a Ag/$AgNO_3$ (0.01 M in CH_3CN) reference electrode under N_2 atmosphere at room temperature. Tetra-*n*-butylammonium hexafluorophosphate (0.1 M in CH_2Cl_2) was used as the supporting electrolyte. The redox potentials were recorded at a scan rate of 100 mV/s and are reported with reference to the ferrocene/ferrocenium (Fc/Fc^+) redox couple.

2. Calculation method

The ground-state geometrical configuration was optimized by density functional theory (DFT) with B3LYP functional. Based on the optimized ground state molecular structure, the time-dependent DFT (TDDFT) approach

associated with the polarized con-tinuum model (PCM) in dichloromethane media was carried out to obtain the vertical excitation energies of triplet states (Tn). The calculation was performed using the Gaussian 16 B.01 suite of programs. The SDD basis set was used to treat the iridium atom, whereas the 6-31G* basis set was used to treat all other atoms. The contours of the highest occupied molecular orbital (HOMO) and lowest unoccupied molecular orbital (LUMO) were plotted by Multiwfn 3.8 soft.

3. Synthesis of cyclometalating ligand and Iridium(III) Complexes

Synthetic routes of **Ir1** and **Ir2** are described in **Figure 1**.

Synthesis of main ligand 1: The synthesis of main ligand 1 is according to our previously reported methods [1].

(4-Chloro-3-(trifluoromethyl)phenyl)boronic acid (1.89 g, 8.4 mmol), 2-chloro-4-methylquinoline (1.5 g, 8.4 mmol), 0.5 g K_2CO_3 and tetrakis(triphenylphosphine)palladium (0.29 g, 0.3 mmol) were added into a 150 mL flask. The mixture system was evacuated under vacuum and flushed with dry nitrogen for three times. Then the degassed toluene (45 mL) was injected. The system was heated to 85°C and stirred for 18 h, and then cooled to room temperature. Next, 50 mL dichloromethane was added into the flask, the system was filtered and the filtrate was collected, then the solvent was removed by rotary evaporator. The crude product was purified by flash chromatography (silica gel, ethyl acetate / petroleum ether, 75/1 to 60/1, v/v),

yielding a yellow solid. ^{1}H NMR (500 MHz, $CDCl_3$, δ: ppm): 8.52 (d, J=2.05 Hz, 1H), 8.28 (dd, J=8.35 Hz, 2.05 Hz, 1H), 8.17 (d, J=8.4 Hz, 1H), 8.02 (dd, J=8.30 Hz, 0.80 Hz, 1H), 7.75 (ddd, J=1.35 Hz, 6.90 Hz, 8.35 Hz, 1H), 7.69 (s, 1H), 7.64 (d, J=8.35 Hz, 1H), 7.59 (ddd, J=1.2 Hz, 6.85 Hz, 8.2 Hz, 1H), 2.79 (d, J=0.8 Hz, 3H).

Synthesis of Iridium(III) Complex:

The μ-dichloro bridged Ir(III) dimer complex 2 was synthesized according to the published literature. [1–3] $IrCl_3·3H_2O$ (1.23 g, 3.5 mmol) and the ligand 1 (2.25 g, 7.0 mmol) were added into a mixture of 2-ethoxyethanol and deionized water (60 mL, 3:1 v/v). Then the system was heated to 110 °C under N_2 atmosphere for 15 h. After the system was cooled, the orange solid was obtained by filtering the solution and then washed with deionized water and methanol. Next, the solid was dried using a vacuum drying oven.

The synthesis of **Ir1** and **Ir2** are according to the previously reported method. [1–3]

Ir1/Ir2: μ-dichloro bridged Ir(III) dimer complex 2 (0.5 g, 0.29 mmol), 4,4'-dimethyl-2,2'-bipyridyl or 4,4'-dimethoxy-2,2'-bipyridyl (0.12 g/0.14 g, 0.60 mmol) were added into a mixture of degassed dichloromethane and methanol (30 mL, 1:1 v/v), the system was stirred at room temperature for 4 h under nitrogen atmosphere. Then the solvent was removed by a rotary evaporator. The crude product was purified by column chromatography using dichloromethane/methanol as eluent.

Ir1: yellow powder (60% yield). ^{1}H NMR (500 MHz, CD_2Cl_2, δ): 8.35 (s, 2H), 8.11 (s, 2H), 8.01~7.97 (m, 4H), 7.94 (s, 2H), 7.51 (t, *J* = 8.20 Hz, 2H), 7.31~7.28 (m, 4H), 7.11 (t, *J* = 7.40 Hz, 2H), 6.99 (s, 2H), 2.97 (s, 6H), 2.51 (s, 6H). ^{13}C NMR (125 MHz, CD_2Cl_2): 166.80, 156.82, 154.98, 152.82, 150.55, 146.87, 146.78, 145.31, 136.66, 133.45, 131.39, 128.91, 128.16, 127.38, 125.57, 125.40 (q, $^3J_{13C\text{-}19F}$= 5.2 Hz), 124.93, 124.80, 123.59 (q, $^1J_{13C\text{-}19F}$= 271.4 Hz), 122.94 (q, $^2J_{13C\text{-}19F}$= 31.2 Hz). ^{31}P NMR (202 MHz, CD_2Cl_2, δ): -144,41 (sep, *J* = 709.12 Hz). ^{19}F NMR (470 MHz, CD_2Cl_2, δ): -62.24 (s), -72.95 (d, *J* = 714.40 Hz). MALDI-TOF-MS (m/z): calcd for $C_{46}H_{32}Cl_2F_6IrN_4$, 1017.15; found, 1017.67.

Ir2: yellow powder (59% yield). ^{1}H NMR (500 MHz, CD_3OCD_3, δ): 8.69 (s, 2H), 8.64 (s, 2H), 8.23 (d, *J* = 6.50 Hz, 2H), 8.14 (d, *J* = 8.30 Hz, 2H), 7.97~7.96 (m, 2H), 7.59 (t, *J* = 7.25 Hz, 2H), 7.53 (d, *J* = 8.90 Hz, 2H), 7.29~7.23 (m, 4H), 6.77 (s, 2H), 3.99 (s, 6H), 3.02 (s, 6H). ^{13}C NMR (125 MHz, acetone-d6): 168.23, 167.36, 161.85, 158.02, 156.98, 150.81, 149.38, 146.88, 146.29, 136.40, 132.53, 131.32, 128.25, 127.37, 126.00 (q, $^3J_{13C\text{-}19F}$ = 5.19 Hz), 125.86, 125.09, 123.81 (q, $^1J_{13C\text{-}19F}$ = 270.01 Hz), 122.29 (q,$^2J_{13C\text{-}19F}$ = 31.1 Hz), 119.31, 114.50, 110.63, 56.41, 18.13. ^{31}P NMR (202 MHz, CD_3OCD_3, δ): -144,26 (sep, *J* = 705.99 Hz). ^{19}F NMR (470 MHz, CD_3OCD_3, δ): -62.08 (s), -72.62 (d, *J* = 709.70 Hz). MALDI-TOF-MS (m/z): calcd for $C_{46}H_{32}Cl_2F_6IrN_4O_2$, 1049.14; found, 1049.69.

References

1. Guo, S.; Guo, C. X.; Lu, Z.; Du, L. L.; Gao, M.; Liu, S. J.; Liu, Y. L.; Zhao, Q. Crystals. 2021, 11, 1190.
2. Sun, H. B.; Liu, S. J.; Lin, W. P.; Zhang, K. Y.; Lv, W.; Huang, X.; Huo, F. W.; Yang, H. R.; Jenkins, G.; Zhao, Q.; Huang, W.. Nat. Commun. 2014, 5, 3601.
3. Nonoyama, M. Bull. Chem. Soc. Jpn. 1974, 47, 767.

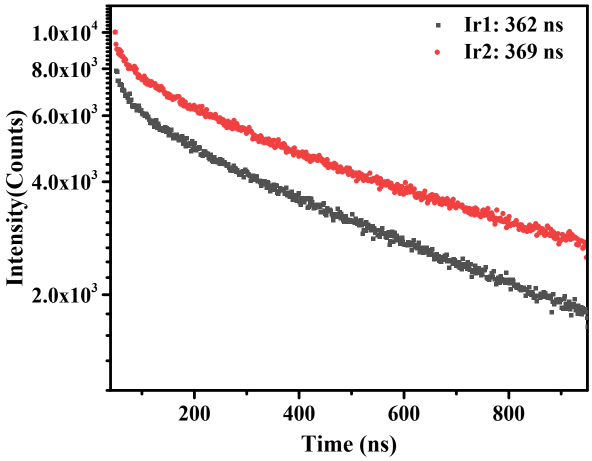

Figure S1. The decay curves of phosphorescent lifetime for **Ir1** and **Ir2** in solid state.

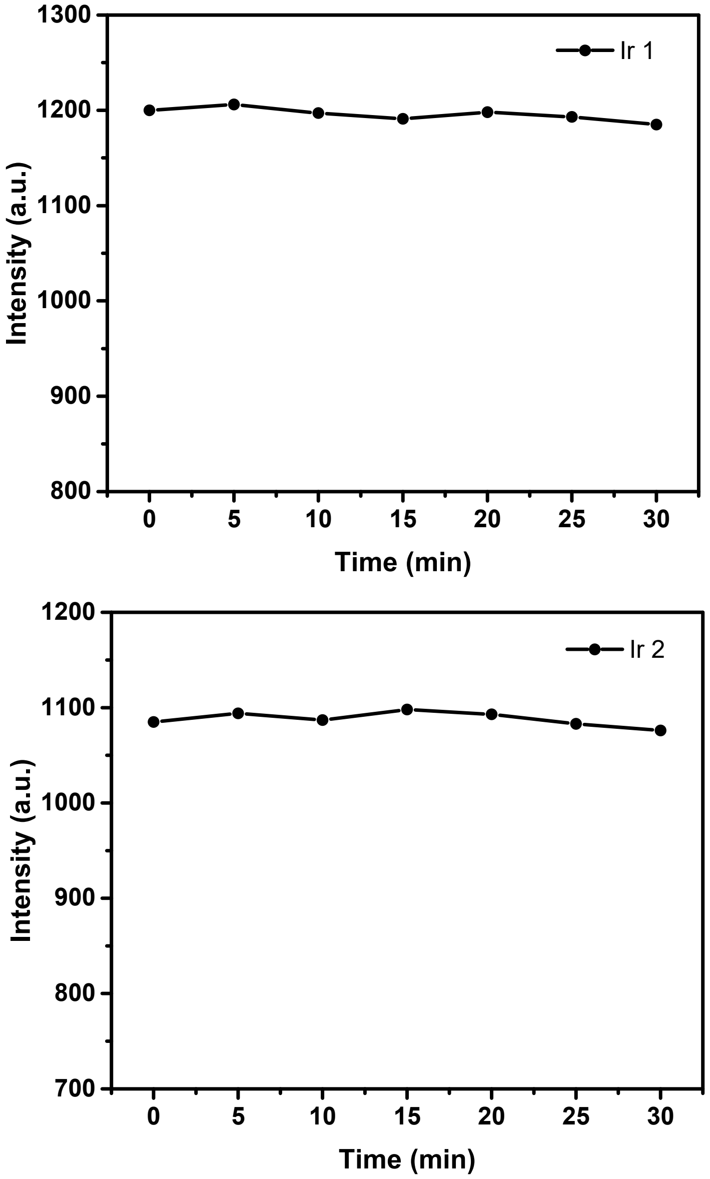

Figure S2. The photostability of **Ir1** and **Ir2** excited at 365 nm in CH_2Cl_2 solution.

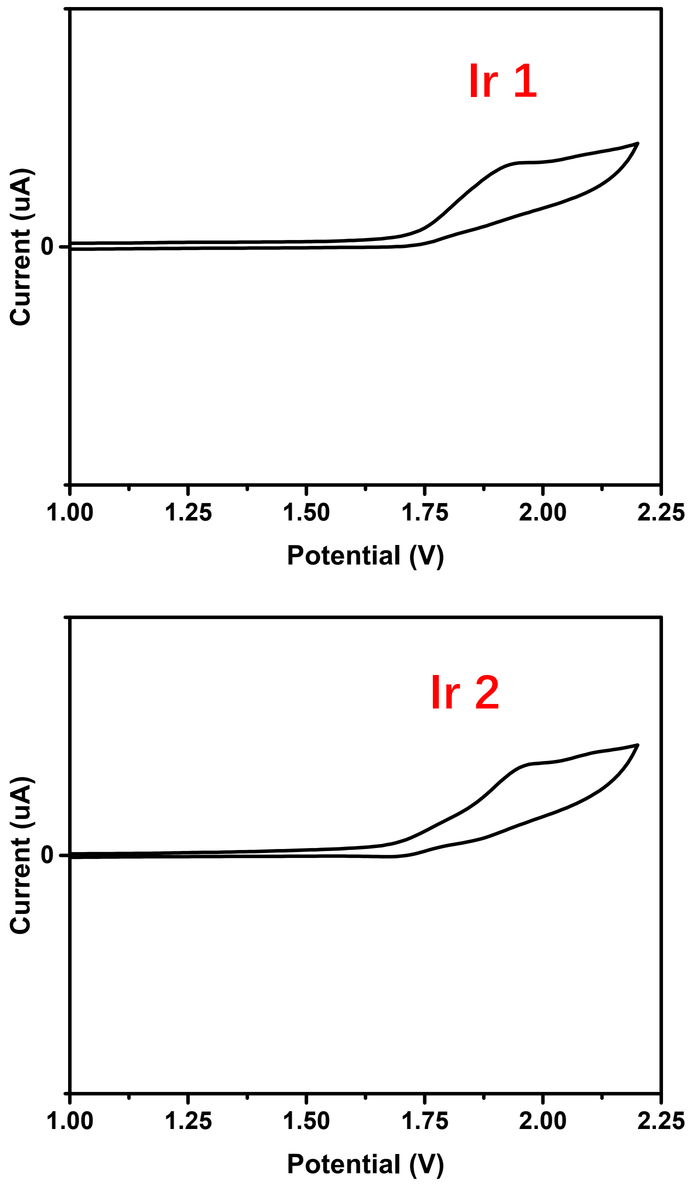

Figure S3. The cyclic voltammograms of **Ir1** and **Ir2** under a scan rate of 100 mV/s in CH_2Cl_2.

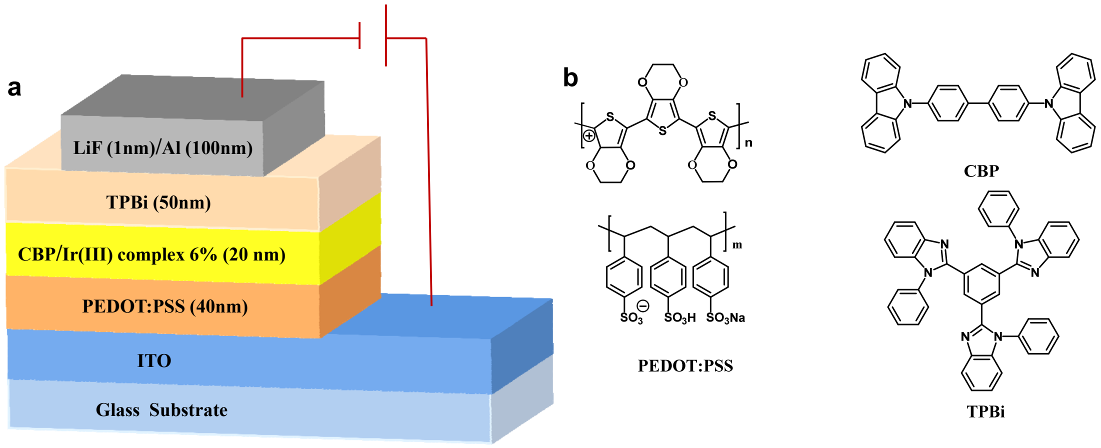

Figure S4. Schematic diagram of device structure (a) of the yellow OLEDs and chemical structures (b) of the materials involved in the prepared devices.

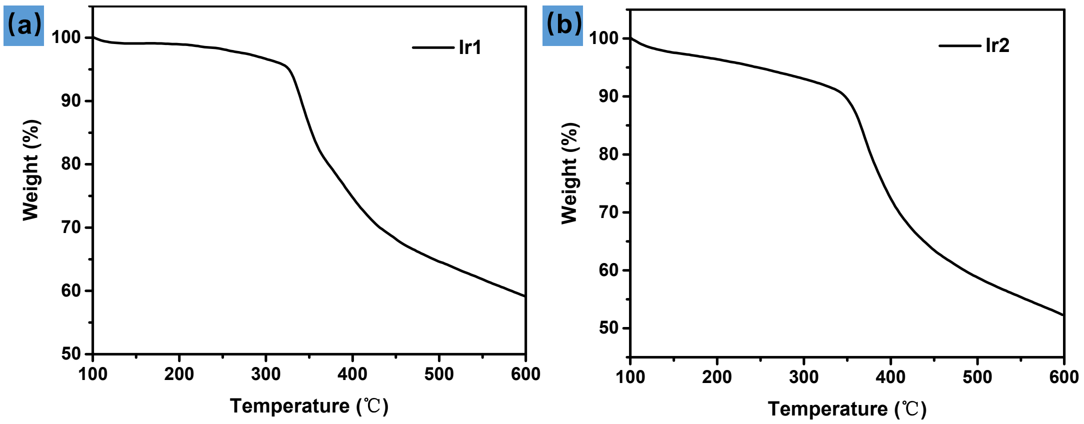

Figure S5. TGA curves of Ir1 and Ir2.

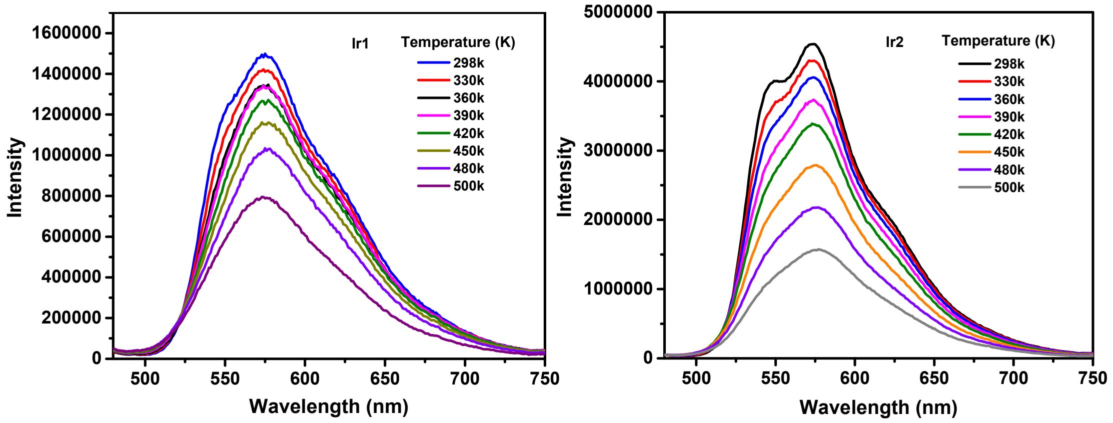

Figure S6. The emission spectra of Ir1 and Ir2 at different temperatures.

NMR and MS Spectra

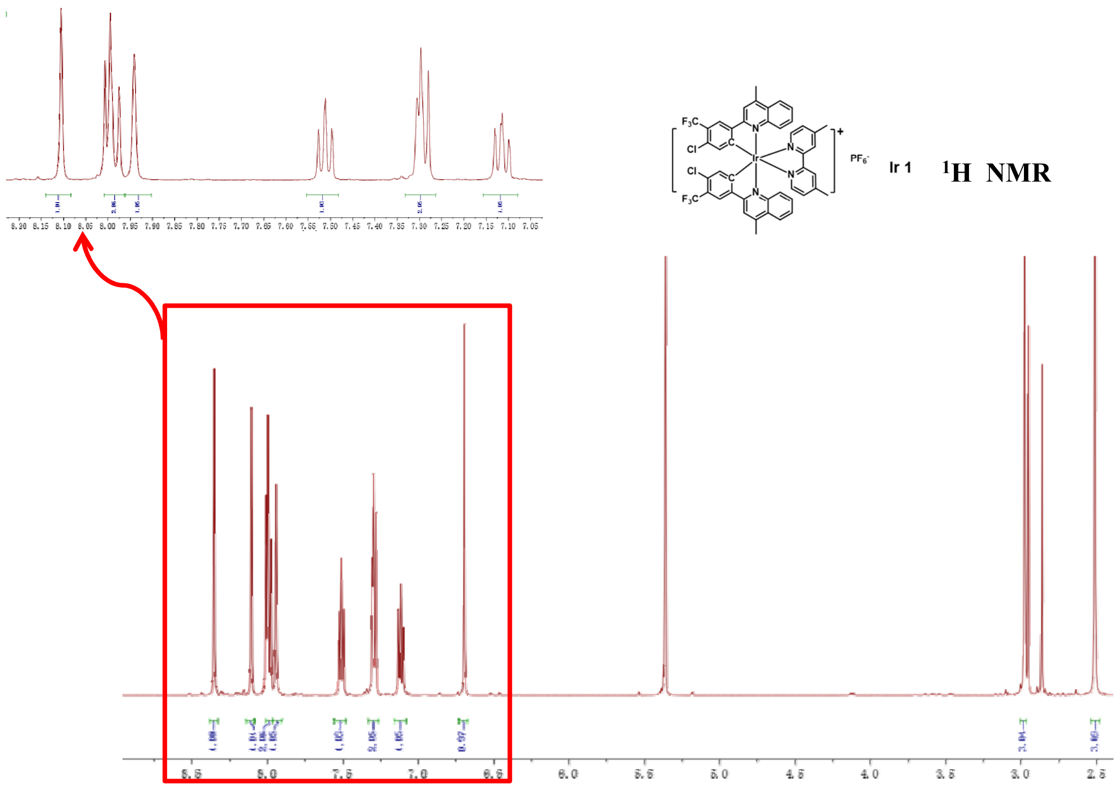

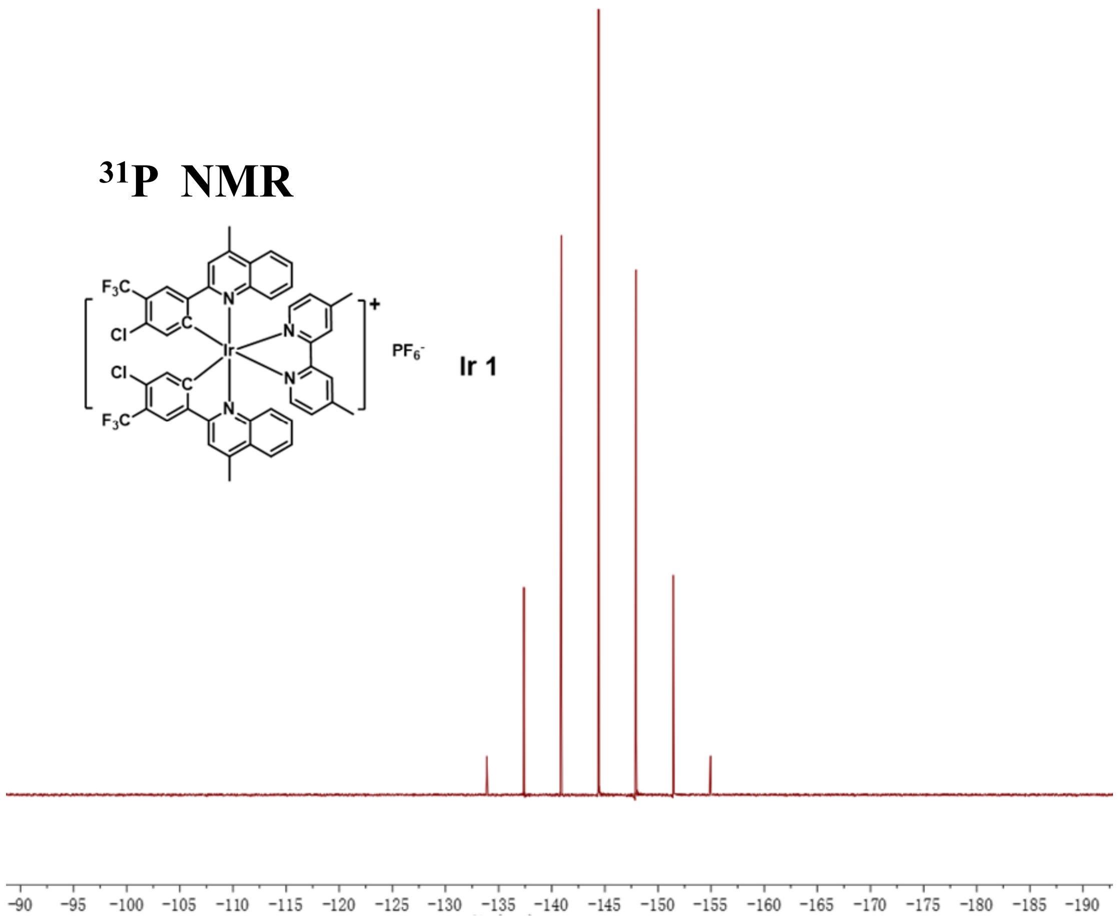

^{31}P NMR
F$_3$C
Cl
C
N
Ir
N
N
+
PF$_6^-$
Ir 1
-90 -95 -100 -105 -110 -115 -120 -125 -130 -135 -140 -145 -150 -155 -160 -165 -170 -175 -180 -185 -190

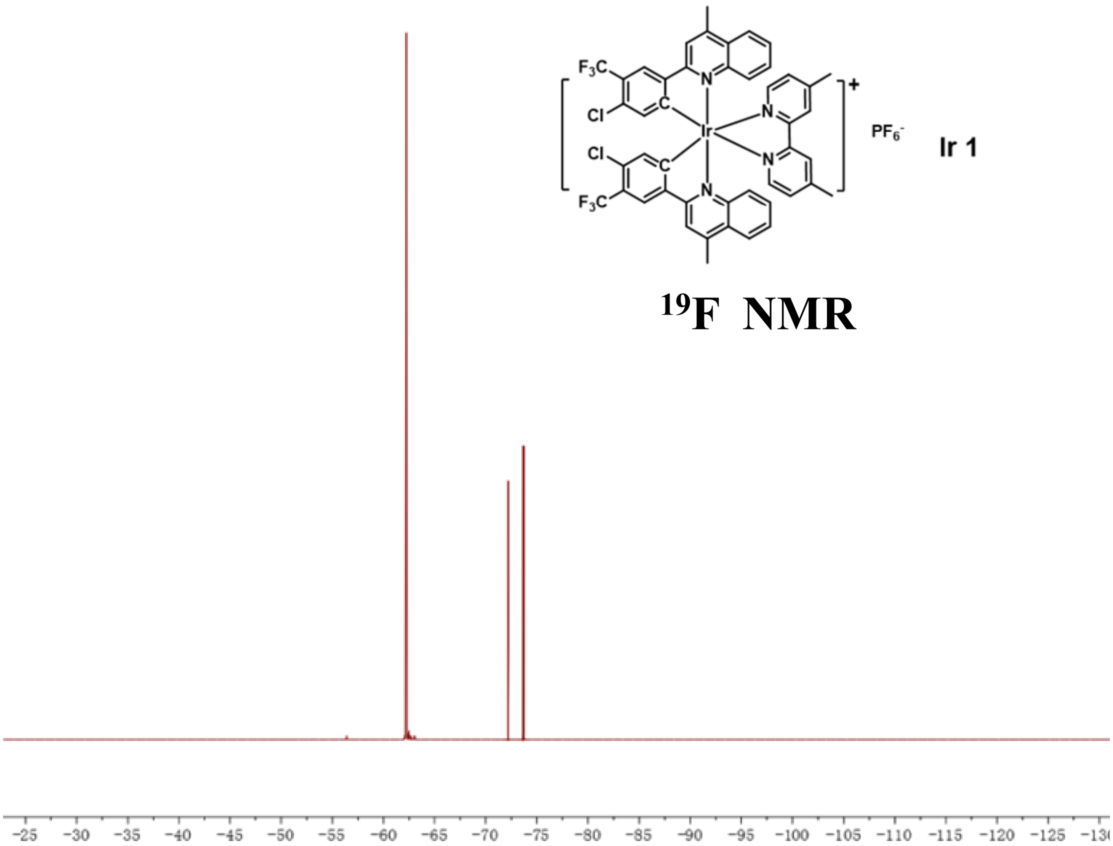
F3C
Cl
Ir
N
PF6-
Ir 1
19F NMR
-25 -30 -35 -40 -45 -50 -55 -60 -65 -70 -75 -80 -85 -90 -95 -100 -105 -110 -115 -120 -125

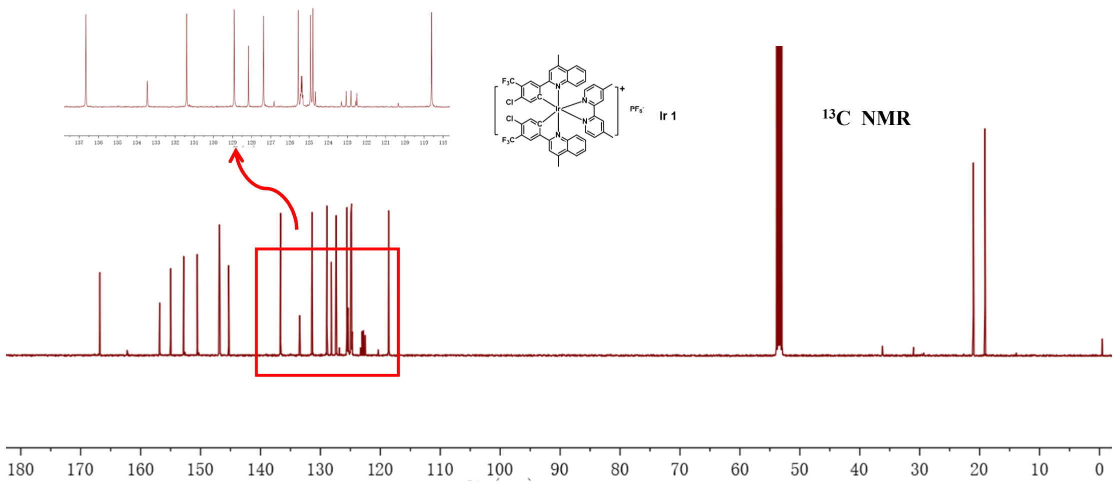

Ir 1
13C NMR
180 170 160 150 140 130 120 110 100 90 80 70 60 50 40 30 20 10 0

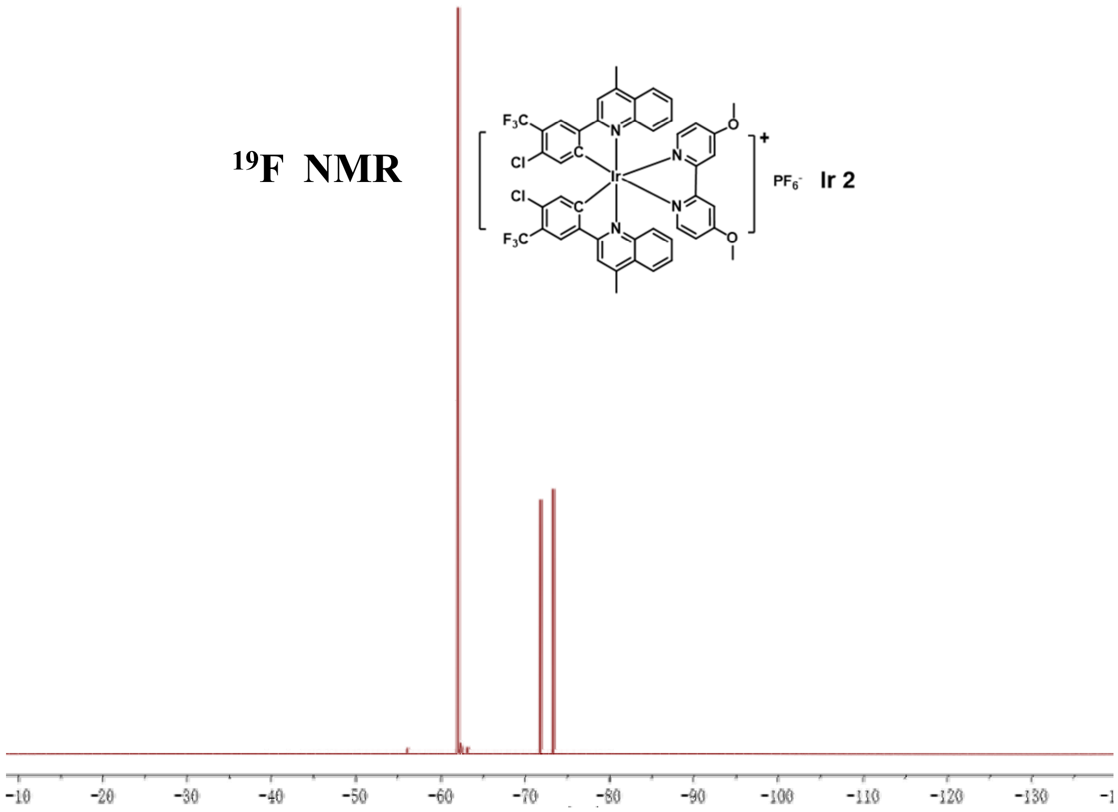

19F NMR
F3C
Cl
C
N
Ir
PF6-
Ir 2
-10
-20
-30
-40
-50
-60
-70
-80
-90
-100
-110
-120
-130

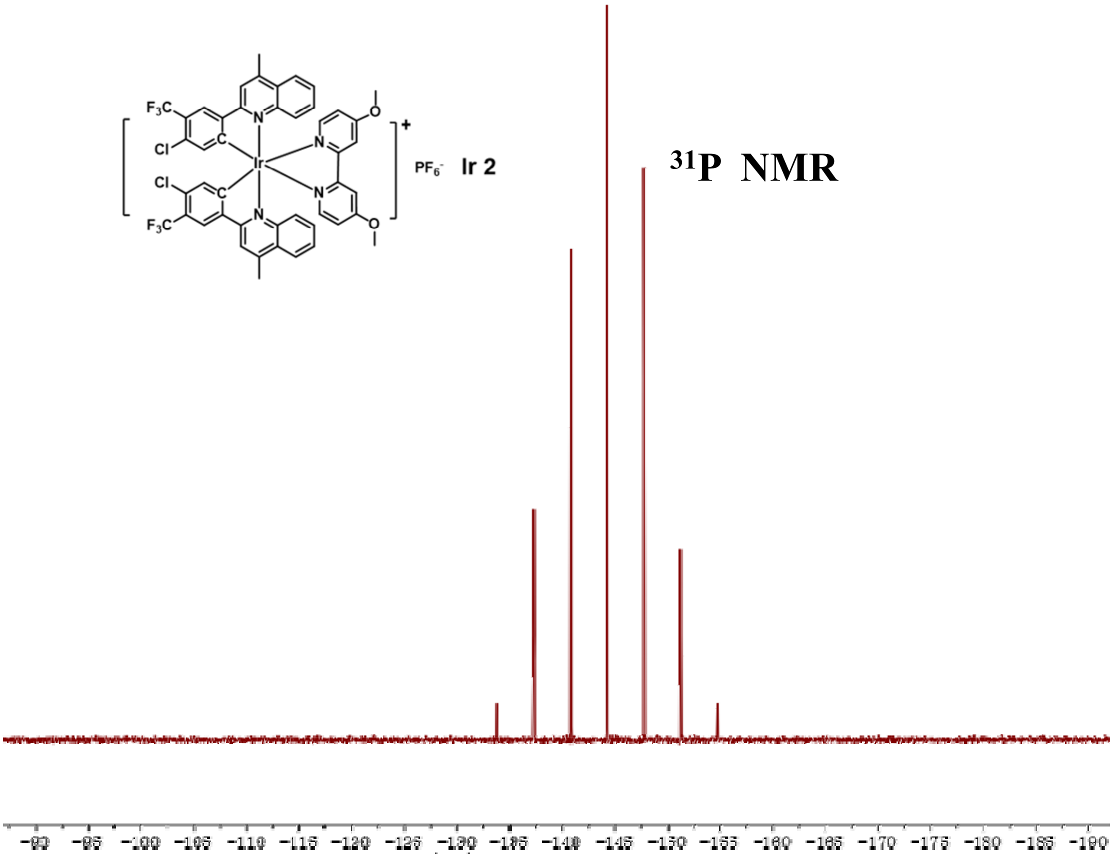
F3C
Cl
Cl
F3C
Ir
N
N
N
N
C
C
O
O
+
PF6-
Ir 2
31P NMR
-90 -95 -100 -105 -110 -115 -120 -125 -130 -135 -140 -145 -150 -155 -160 -165 -170 -175 -180 -185 -190

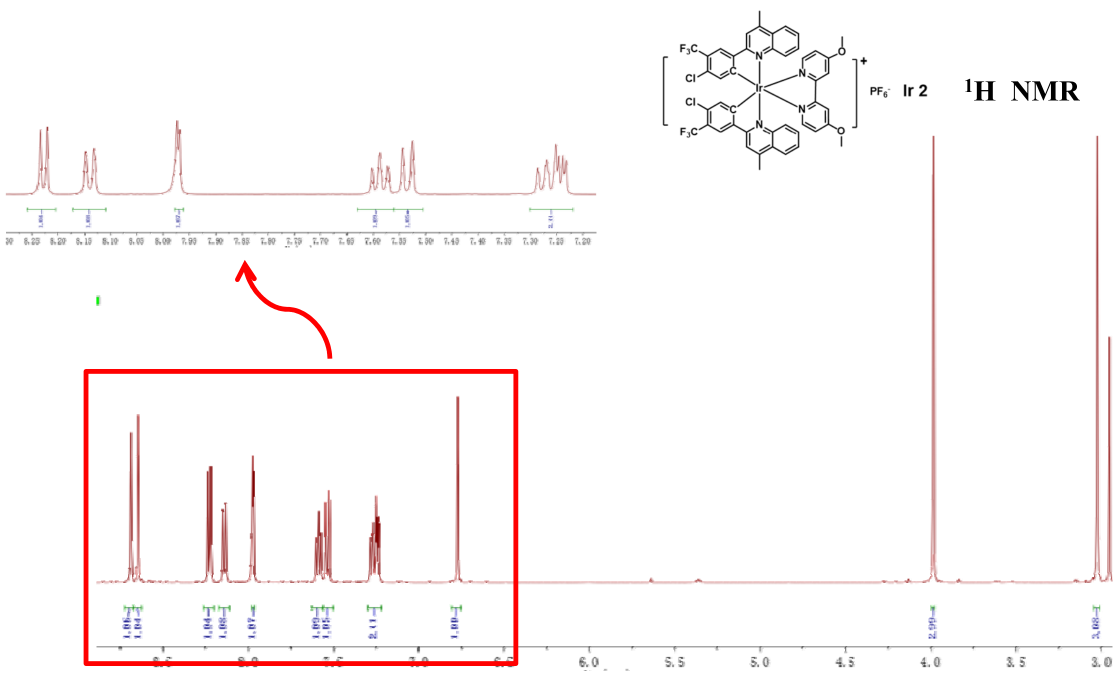

Ir 2
^{1}H NMR
PF_6^-

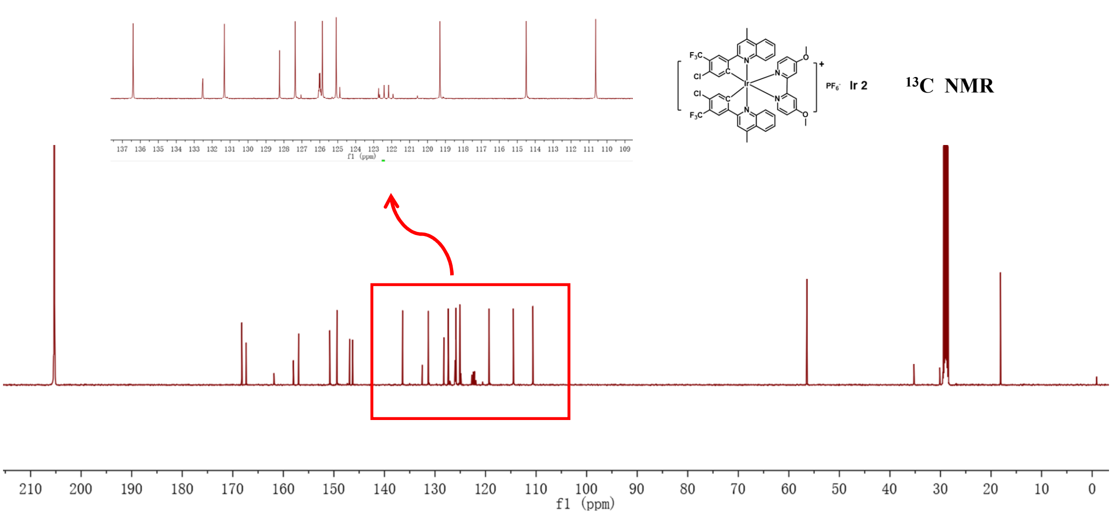

Ir 2
13C NMR
210 200 190 180 170 160 150 140 130 120 110 100 90 80 70 60 50 40 30 20 10 0
f1 (ppm)

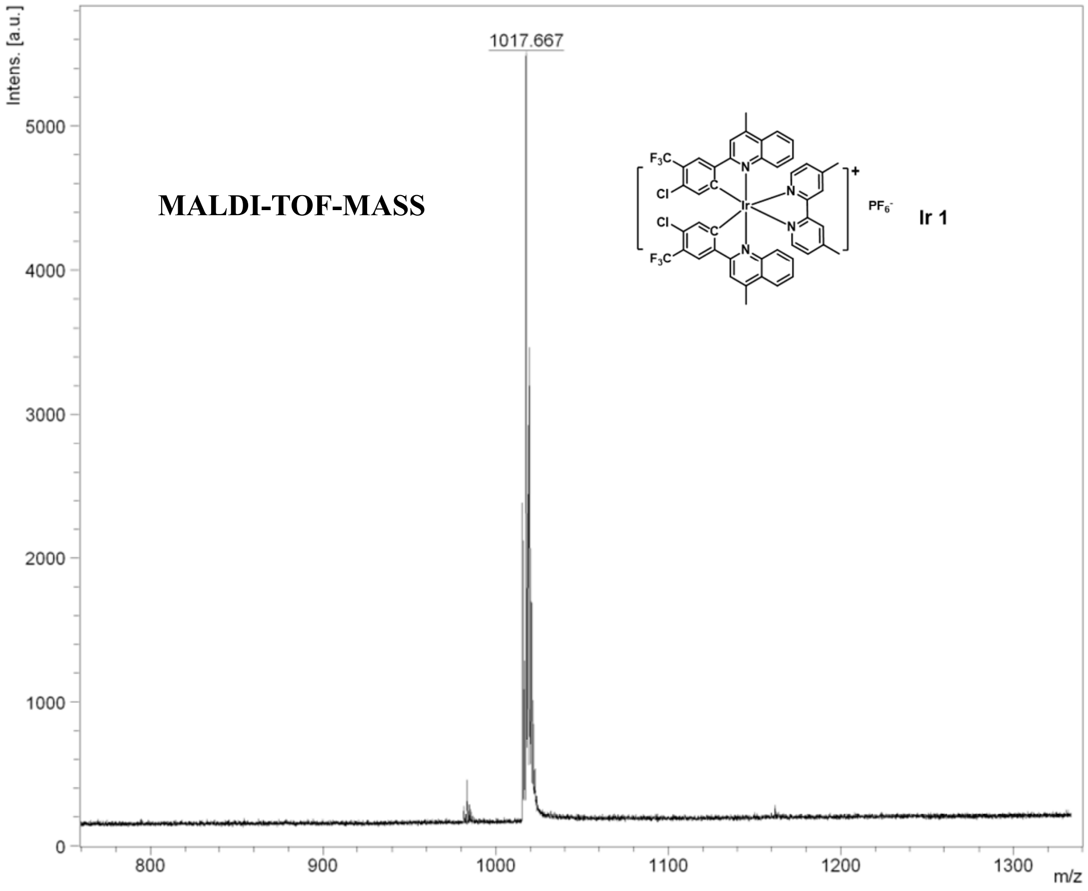

Intens. [a.u.]
1017.667
MALDI-TOF-MASS
Ir 1
PF6-
0
1000
2000
3000
4000
5000
800
900
1000
1100
1200
1300
m/z

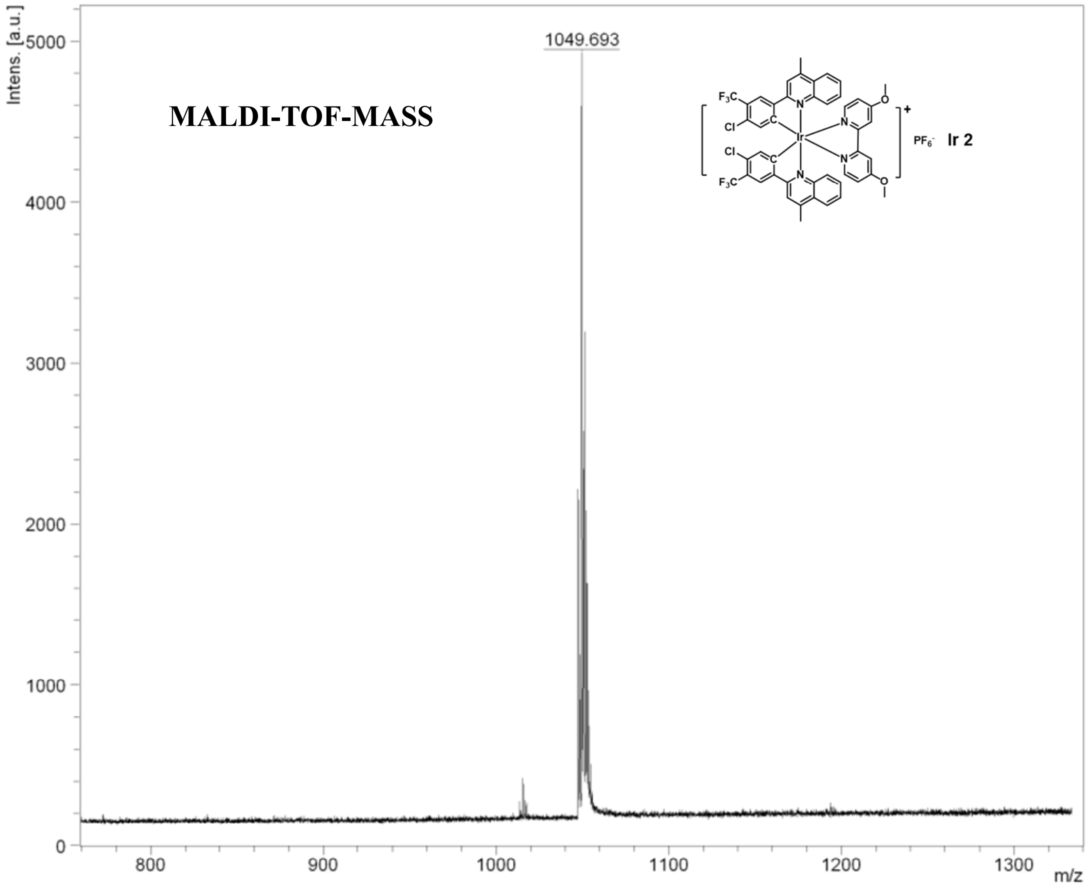

Intens. [a.u.]
5000
4000
3000
2000
1000
0
1049.693
MALDI-TOF-MASS
F3C
Cl
Cl
F3C
N
C
Ir
N
N
N
C
O
O
+
PF6-
Ir 2
800
900
1000
1100
1200
1300
m/z
